# Molecular Subtyping of Serous Ovarian Cancer Based on Multi-omics Data

**DOI:** 10.1038/srep26001

**Published:** 2016-05-17

**Authors:** Zhe Zhang, Ke Huang, Chenglei Gu, Luyang Zhao, Nan Wang, Xiaolei Wang, Dongsheng Zhao, Chenggang Zhang, Yiming Lu, Yuanguang Meng

**Affiliations:** 1 Department of Gynecologic Oncology, Chinese PLA General Hospital, Beijing 100853, China; 2 Beijing Institute of Radiation Medicine, State Key Laboratory of Proteomics, Cognitive and Mental Health Research Center, Beijing 100850, China; 3 Beijing Institute of Health Service and Medical Information, Beijing 100850, China

## Abstract

Classification of ovarian cancer by morphologic features has a limited effect on serous ovarian cancer (SOC) treatment and prognosis. Here, we proposed a new system for SOC subtyping based on the molecular categories from the Cancer Genome Atlas project. We analyzed the DNA methylation, protein, microRNA, and gene expression of 1203 samples from 599 serous ovarian cancer patients. These samples were divided into nine subtypes based on RNA-seq data, and each subtype was found to be associated with the activation and/or suppression of the following four biological processes: immunoactivity, hormone metabolic, mesenchymal development and the MAPK signaling pathway. We also identified four DNA methylation, two protein expression, six microRNA sequencing and four pathway subtypes. By integrating the subtyping results across different omics platforms, we found that most RNA-seq subtypes overlapped with one or two subtypes from other omics data. Our study sheds light on the molecular mechanisms of SOC and provides a new perspective for the more accurate stratification of its subtypes.

Ovarian cancer is the eighth-leading cancer type worldwide and the leading cause of gynecological cancer-related death among women^1^. It is considered to have the worst prognosis of all gynecological malignant tumors, causing 140,200 deaths each year^2^,^3^. Serous ovarian cancer (SOC) is the most common subtype of epithelial ovarian cancer and accounts for approximately 85% of ovarian neoplasms^1^. Currently, SOC treatment mainly relies on surgical resection, which is assisted by chemotherapy, targeted therapy, hormone therapy, and radiotherapy^1^,^4^,^5^. Drug therapy for SOC is most often a combination of taxane and platinum, and targeted drugs are also being assessed, including bevacizumab and olaparib^2^,^3^,^4^. Targeted therapy has been highly anticipated, but it did not have a revolutionary effect on the comprehensive treatment for SOC. Although SOC has been classified by many categories, including histopathology, FIGO (International Federation of Gynecology and Obstetrics) stage and tumor grade, the actual significance of precision therapy or personalized therapy in cancer treatment is still far from ideal. The existing traditional and histological classification of ovarian cancer is of limited prognostic significance. High mortality rates, low early detection rates, and a lack of reliable biomarkers and effective molecular classification for prognosis make SOC more complex than other gynecological cancers^6^.

Studies on ovarian cancer subtyping are expected to shed light on the understanding of the molecular mechanisms of this disease, aid in the development of more specific therapies, and identify novel genetic and environmental risk factors^7^. Recent studies demonstrated that environmental and genetic risk factors, multiple somatic mutations, and clinical response rates to platinum/taxane-based therapy varied substantially between the histotypes of ovarian cancer^8^. However, due to the high cost of large-scale molecular profiling, prior research only focused on a small number of genes or single-platform genomic data^9^, such as gene expression data^7^ or DNA methylation data^10^,^11^,^12^. Fortunately, the Cancer Genome Atlas (TCGA) project has published many cancer samples (including SOC) with publically available multi-omics data^8^, allowing researchers to investigate cancer subtyping across different omics data. Nevertheless, the super-high dimensional omics data has introduced a great challenge to statistical modeling of data. A systemic approach for selecting biologically important genetic variables and classifying cancer samples based on multi-omics data is still needed to be improved. In addition, little attempt has been made to study the effects of the number of subtypes on the classification algorithm.

The classification of serous ovarian cancer by surgical-pathological staging has helped guide clinical practice; however, it has added little prognostic or predictive information for clinical decisions^13^. Here, we develop a meaningful computational framework for the molecular classification of ovarian cancer based on multi-omics data. We evaluate a series of unsupervised classification algorithms with different numbers of subtypes. We then investigate the association between molecular subtypes and prognosis as well as a serial of clinical factors, including surgery, pathology, chemotherapy, radiotherapy, recurrence, and follow-up outcomes^8^. We integrate the subtyping results by different omics data and assess their relationships. We anticipate our work can contribute to the understanding of SOC and provide a new perspective for a more accurate stratification.

## Results

### A systemic framework for SOC subtyping on the basis of multi-omics data

The multi-omic SOC dataset from the TCGA project contains a total of 1203 samples that were collected from tumor tissues, adjacent normal tissues or blood samples of 599 SOC patients. The TCGA cohorts also contained information on the overall survival time and clinical variables (e.g., age, gender, drugs and tumor stage). We obtained four types of molecular data that are available for open access: (i) DNA methylation: Illumina DNA Methylation microarray, ~20,000 genes; (ii) protein expression: reverse-phase protein array, ~170 proteins; (iii) microRNA (miRNA) expression: Agilent Human miRNA-specific microarray or Illumina miRNA-seq, >500 microRNAs; and (iv) mRNA expression: Agilent 244 K microarray or Illumina mRNA-seq, ~20,000 genes. Table 1 lists the platforms of multiple molecular detection data from TCGA. We focused on a core SOC sample set in which each sample has information available for the survival time and at least one out of the four types of molecular data (Figure S1). In our dataset, in terms of the follow-up date, survivals accounted for 46.9% (281/599), deaths accounted for 50.4% (302/599), and missing data for 2.7% (16/599). With respect to the treatment outcome of the first course, patients with complete remission accounted for 55.4% (332/599), partial remission 10.9% (65/599), stable disease 5.7% (34/599), progressive disease 7.5% (45/599), and missing data 20.5% (123/599).

Feature selection is necessary to improve the robustness of clustering. We used a Cox regression model to select biologically important features from all of the available genomic features in the dataset^14^,^15^. For each genomic feature, we examined its predictive power for the survival time of patients after their initial diagnoses, and features that achieved significant predictive models (*P*-value < 0.05) were selected to constitute the final feature set for further classification. In addition, we developed a novel method for reducing the feature dimensions without using the patient survival data. Specifically, we converted the gene expression levels to the activity levels of 137 KEGG pathways, which were then used as input features for unsupervised clustering. This pathway level-based method classified patients into cancer subtypes without any a priori knowledge of patient survival information, which is a good alternative for molecular level-based subtyping methods.

For each molecular data type, we classified the SOC samples into subtypes using the following three unsupervised clustering methods: Partitioning Around Medoids (PAM)^16^,^17^, Hierarchical Clustering (HC)^18^ and Non-Negative matrix Factorization (NMF)^19^. To evaluate the performance of different clustering models, we performed Kaplan-Meier survival analysis of the patients were classified into difference clusters, and the significance of the survival analysis was used to evaluate the performance of the subtyping model^20^. Meanwhile, the increase in the model complexity (number of clusters) will enhance the performance of survival analysis, while harming the interpretability of the model. As a result, we defined a model complexity penalized score to determine the optimal model that achieves the best balance between clinical significance and model complexity (see Methods and Materials). To determine the optimal number of subtypes in each dataset, we carefully screened the number of clusters from 2 to 9, and the model with the highest penalized score was selected as the optimal model.

### SOC subtyping based on RNA-seq

**Figure 1:**
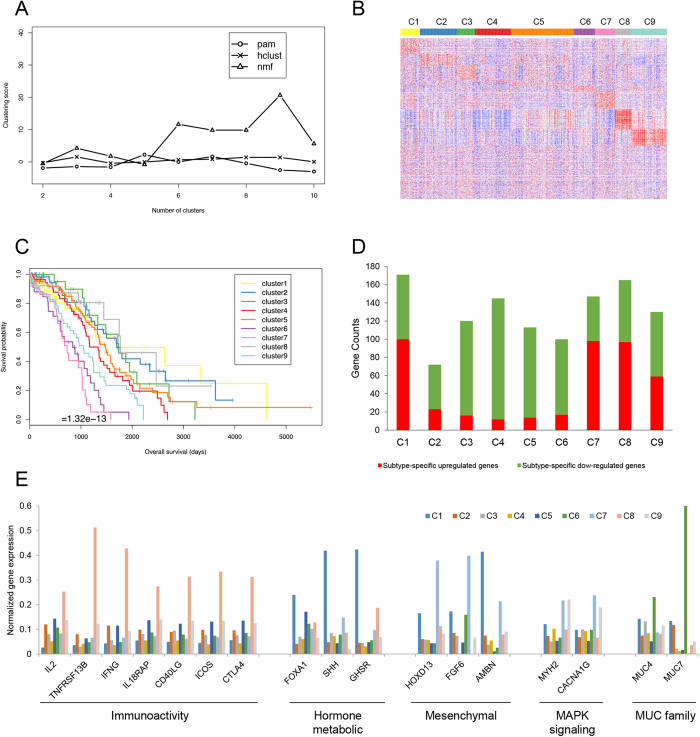
RNA sequencing data of (**A**) compared three different unsupervised clustering methods, (**B**) heatmap of 1384 significant genes among nine subtypes. Rows were ordered by their significance of up-regulation in each cluster. Genes not significantly up-regulated in any cluster were moved to the end of the map. (**C**) Survival curves of nine subtypes, (**D**) counts of subtype-specific up-regulated genes and down-regulated genes. Up-regulated and down-regulated genes were determined by the thresholds of 2-fold and 0.5-hold change. (**E**) Average gene expression levels of representative genes of the four biological processes in nine RNA-seq subtypes.

The SOC RNA-seq dataset consisted of the mRNA expression levels of ~22,000 genes. A total of 420 primary solid tumor cohort samples with both genomic and survival data were used by excluding unavailable sequencing data and the recurrent solid tumor cohort. We identified 1384 genes that were significantly related to patient survival (*P*-value < 0.05, Cox regression analysis). We performed the three clustering algorithms (PAM, HC and NMF) using the 1384 significant genes and found NMF with eight subtypes achieved the highest score (Fig. 1A). The survival curve and heatmap are shown in Fig. 1B,C. The number of patients in the nine subtypes were: C1 (n = 31, median survival = 1720 days), C2 (59, 1699 days), C3 (28, 1736 days), C4 (56, 1169 days), C5 (100, 1470 days), C6 33, 840 days), C7 (32, 730 days), C8 (25, 1767 days) and C9 (56, 1046 days).

Using the expression profiles of 1384 genes across the nine SOC subtypes, we defined two sets of genes that were associated with each subtype respectively – subtype-specific up-regulated genes and subtype-specific down-regulated genes (see Methods and Materials). The counts of genes in the two gene sets of different SOC subtypes are shown in Fig. 1D (Supplementary Table S1). For each set of subtype-specific up-regulated and down-regulated genes, we performed Gene Ontology and KEGG pathway enrichment analysis (Supplementary Tables S2–S10). Interestingly, we found that these gene sets were mainly enriched in the following four biological processes: immunoactivity, hormone metabolic, mesenchymal development and MAPK signaling (Table 2). Genes associated with nine subtypes displayed distinct expression profiles among the four biological processes. Table 2 summarizes the gene expression profiles of the nine SOC subtypes across the four biological processes and a set of reported SOC biomarkers: the *MUC* gene family^21^. For each of the four biological processes and the *MUC* gene family, we identified a number of gene markers that were frequently presented in the subtype-specific gene sets. The immunoactivity-related markers included *IL2, TNFRSF13B, IFNG, IL18RAP, CD40LG, ICOS* and *CTLA4*; the hormone metabolic-related markers included *FOXA1, SHH* and *GHSR*; the mesenchymal-related markers included *HOXD13, FGF6* and *AMBN*; the MAPK signaling pathway-related markers included *MYH2* and *CACNA1G*; and the *MUC* family markers included *MUC4* and *MUC7*. Their expression profiles across the nine SOC subtypes are shown in Fig. 1E.

We also analyzed the differences of a total of 18 clinical variables among the nine SOC subtypes. These clinical variables included the tumor size, lymphovascular invasion, diagnosis days, new tumor event days, tumor grade, clinical stage, and so on. The clinical stage was obtained from clinical information on the tumor size, extent of the primary tumor and whether the tumor spread to other parts of the body. The tumor grade was used to assess the degree of differentiation that was related to the clinical behavior and used to classify cancer cells as G1, G2, G3 or G4. The tumor grade is the histology description based on NCI (https://ncit.nci.nih.gov/), while the clinical stage was based on the AJCC (American Joint Committee on Cancer) staging criteria. The eight quantitative variables were analyzed using one-way ANOVA (analysis of variance) and another 10 qualitative variables were analyzed using the chi-square test for one-way tables (Table 3). We found that the clinical stage (P = 1.22 × 10^−5^) and treatment outcome from the first course (P = 0.03) exhibited significant difference across nine SOC subtypes, while the presence of a vascular (P = 0.477) or lymphoma invasion (P = 0.528) and the tumor grade (P = 0.169) showed no significant difference among those subtypes.

### SOC subtyping based on DNA methylation, protein and microRNA expression

#### DNA methylation array

**Figure 2:**
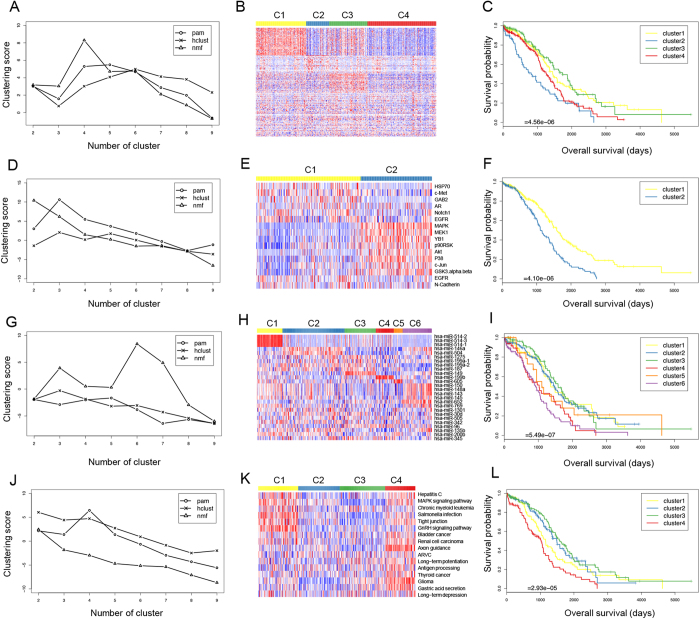
DNA methylation of (**A**) three different unsupervised clustering methods, (**B**) heatmap of 201 CpG sites among four subtypes, and (**C**) survival curves of four subtypes. Protein expression of (**D**) three different unsupervised clustering methods, (**E**) heatmap of 16 proteins among two subtypes, and (**F**) survival curves of two subtypes. MiRNA expression (based on the sequencing platform) of (**G**) three different unsupervised clustering methods, (**H**) heatmap of 38 miRNAs among six subtypes, and (**I**) survival curves of six subtypes. Pathways of (**J**) three different unsupervised clustering methods, (**K**) heatmap of 16 pathways among four subtypes, and (**L**) survival curves of four subtypes. Rows in the heatmaps were ordered by their significance of up-regulation in each cluster. Features not significantly up-regulated in any cluster were moved to the end of the map.

The DNA methylation dataset of SOC in TCGA contains 14,877 CpG sites from 1203 samples. DNA methylation data used was the calculated beta values mapped to genome, per sample. After excluding samples from the recurrent solid tumor cohort and normal solid tissue cohort as well as unavailable data, we selected 583 primary solid tumor cohort samples. The survival information for the corresponding patients were also obtained from the TCGA clinical dataset. By excluding patients whose survival information was unavailable, we selected a total of 568 samples with both genomic and survival data for the further feature selection and clustering procedures. We associated the DNA methylation intensities of 14,877 CpG sites with the survival time of the patients and identified 201 sites that were significantly associated with patient survival (*P*-value < 0.05, Cox regression analysis). To annotate the 201 CpG sites, we mapped these sites to the human genome, and the genes closest to these sites were obtained. We then applied the three unsupervised clustering algorithms to these tumor samples on the basis of the methylation levels of the 201 CpG sites. For each algorithm, we sought to find the optimal number of clusters by screening a range of clusters from 2 to 9. We found that the NMF model with four clusters achieved the highest penalized score among all models (Fig. 2A). The number of patients in the four subtypes were as follows: C1 (n = 160, median survival = 1451 days), C2 (71, 820 days), C3 (121, 1736 days) and C4 (216, 1266 days). The DNA methylation profiles of the 201 CpG sites among the four subtypes of SOC samples are shown in the Fig. 2B, and the survival curves of four subtypes are shown in Fig. 2C. We found that both the DNA methylation profiles and survival curves clearly distinguished among the four groups of patients. Interestingly, we found the global methylation level of the 201 CpG sites correlated with patient survival. Compared to subtypes C2 and C4, subtypes C1 and C3 showed higher global methylation levels at the 201 CpG sites and patients obtained better survival outcomes.

#### Protein array

The SOC protein array dataset in TCGA contains the expression profiles of 165 proteins that were detected by a reverse-phase protein array platform in 1203 samples. After excluding the samples from the recurrent solid tumor and normal solid tissue cohorts as well as patients whose survival information was unavailable, we selected a total of 406 samples with both genomic and survival data. We conducted Cox regression analysis and found that 16 proteins were significantly related to patient survival (*P*-value < 0.05, Cox regression analysis), including MAPK, MEK, YB1, EGFR (two fragments), HSP70, c-Met, p90RSK, Akt, N-Cadherin, p38, GAB2, AR, Notch1, c-Jun, and GSK3. MAPK/ ERK signaling pathway was reported to be associated with ovarian cancer ascites development and carboplatin resistance^22^. YB1 was correlated with resistance and progression to chemotherapy in epithelial ovarian cancer^23^. MEK, EGFR, HSP, c-met, and GAB2 were directly related to ovarian cancer^24^,^25^,^26^,^27^,^28^,^29^, while p90RSK and Akt were indirectly associated with ovarian cancer^30^,^31^. Specifically, the experimental results suggested that melatonin enhanced cisplatin-induced apoptosis via inactivation of the ERK/p90RSK/HSP27 cascade in SK-OV-3 cells (ovarian cancer cell line) as a potent synergist to cisplatin treatment^30^. Additionally, Cav-1 could promote the chemoresistance of ovarian cancer by targeting apoptosis through the Notch1/Akt/NF-κB pathway^31^. Using the expression profiles of the 16 proteins as input features, we performed unsupervised clustering followed by survival analysis. We found that the penalized scores were very close between NMF, with two clusters, and PAM, with three clusters (Fig. 2D). We finally chose the NMF model with two clusters considering that the survival curves and heatmaps of different clusters were better separated (Fig. 2E,F). The number of patients in the two subtypes were as follows: C1 (n = 240, median survival = 1583 days) and C2 (166, 1106 days). We found that protein expression of EGFR (pY992.R.V), HSP70, c-Met, GAB2, AR and Notch1 was higher in subtype C1 and lower in C2, while the expression of MAPK, p38, MEK, YB1, p90RSK, Akt, c-Jun and GSK3 was opposite (Fig. 2E). The survival analysis indicated that high expression of EGFR (pY992.R.V), HSP70, c-Met, GAB2, AR and Notch1 might be beneficial to SOC patients survival while the other eight genes might have the opposite effect (Fig. 2F).

#### MicroRNA sequencing

The SOC microRNA (miRNA) sequencing dataset contained the expression profiles of 705 miRNAs. The unavailable sequencing data and recurrent solid tumor cohort were excluded, resulting in 473 primary solid tumor cohort samples with both genomic and survival data available. We identified 26 miRNAs that were significantly related to patient survival (P < 0.05, Cox regression analysis). Using these miRNAs as input features, we performed sample clustering with the three methods, and their performances are shown in Fig. 2G. We finally chose a NMF model with six clusters. The number of patients in the six subtypes were as follows: C1 (69, 1364 days), C2 (168, 1470 days), C3 (85, 1583 days), C4 (48, 1024 days), C5 (24, 1103 days), and C6 (79, 914 days). The expression profiles for the 26 miRNAs among the six subtypes of SOC samples are shown by heatmap in Fig. 2H, and the survival curves are shown in Fig. 2I. There were three high expressed miRNAs in subtype C1 (Fig. 2H), which were miR-514-1, miR-514-2 and miR-514-3. miR-514 was reported in a study on renal cell carcinoma; its downregulation was related to recurrence and poor prognosis^32^. Reduction of miR-150 could promote the development of epithelial ovarian cancer^33^. In addition, to systemically investigate the potential regulatory function of these microRNAs, we conducted miRNA target genes functional enrichment of their significantly enriched biological processes and pathways using DIANA-mirPath software^34^ (see Methods and Materials). We found various biological processes and pathways were associated to different microRNA sets (Supplementary Table S11). Specifically, among these highly expressed miRNAs in six groups, 14 miRNAs in cluster C1, C2 and C3 was associated with a better prognosis, while the other 12 miRNAs in cluster C4 to C6 might correlate with a poor prognosis in SOC patients (Fig. 2I).

### SOC subtyping based on integrated pathways

To classify the SOC samples at the pathway level, we first converted the expression profile of ~22,000 genes in each sample to the activity information for 137 KEGG pathways (see Methods). We obtained the activity information of 428 SOC samples that had both gene expression and patient survival data. We associated the activities of the 137 pathways with the survival time of the patients and identified 16 pathways that were significantly associated with patient survival (*P*-value < 0.05, Cox regression analysis). These pathways are involved in bladder cancer, hepatitis C, renal cell carcinoma, thyroid cancer, glioma and chronic myeloid leukemia. Our study indicated that the stress response of the MAPK signaling pathway plays an important role in the survival of patients with SOC, which was consistent with a previous observation in a protein array. Immunoregulation of antigen processing also affected the survival time of SOC patients. Unsupervised classification and survival analyses were conducted according to the activity information of 16 pathways. We found that the PAM model with four clusters achieved the highest penalized score among all other models (Fig. 2J). The number of patients in the four subtypes were as follows: C1 (n = 107, median survival = 1158 days), C2 (111, 1492 days), C3 (122, 1579 days), and C4 (80, 1024 days). The activities of the 16 pathways among four subtypes of SOC samples are shown by heatmap in Fig. 2K, and the survival curves of four subtypes are shown in Fig. 2L.

### Integrated analysis of SOC subtypes based on individual omics data

**Figure 3:**
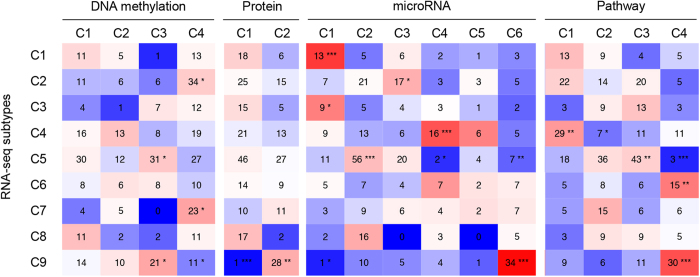
Overlaps between the omics subtypes (the SOC subtypes of DNA methylation, protein, microRNA expression or pathway activity) and nine RNA-seq subtypes. *P < 0.05, **P < 0.01, ***P < 0.001.

We conducted an integrated analysis of different SOC subtyping by RNA-seq, DNA methylation, protein array, miRNA-seq and pathway activity. We investigated the distributions of the SOC subtypes based on each individual omics data as follows: DNA methylation (4 groups), protein array (2 groups), miRNA-seq (6 groups), and pathway activity (4 groups) across nine SOC subtypes generated by RNA-seq data. Specifically, we overlapped the nine RNA-seq SOC subtypes with subtypes by DNA methylation, protein, microRNA expression or pathway activity respectively and found that most of these subtypes overlapped with one or two specific RNA-seq subtypes (Fig. 3). We conducted in-depth analysis to these significantly overlapped subtypes between RNA-seq and the following platforms.

For DNA methylation, cluster C3 in DNA methylation significantly overlapped with cluster C5 and C9 in RNA-seq (both *P*-value < 0.05, Chi-squared test), and cluster C4 in DNA methylation overlapped with cluster C2, C7 and C9 in RNA-seq datasets (both P-value < 0.05) (Fig. 3). We found that mesenchymal development-associated gene *EFNB1* and MAPK-associated genes *FPR1, MAPK7* and *FPR1* was hypermethylated in DNA methylation subtype C3, while they were hypomethylated in subtype C4. These results were consistent with the overlapping between DNA methylation subtypes C3 with RNA-seq subtype C5 (downregulation of mesenchymal development and MAPK pathway) and C9 (upregulation of MAPK pathway). C7 (upregulation of mesenchymal development), and C9 (upregulation of MAPK pathway) (Table 2).

For microRNA-seq, five out of six clusters overlapped with subtypes in RNA-seq. For instance, miRNA cluster C3 overlapped with RNA-seq cluster C2 (P-value < 0.05), and miRNA cluster C6 overlapped with RNA-seq cluster C9 (P-value < 0.001) (Fig. 3). MiRNAs in subtype C4 were predicted to be related with downregulation of immune activity, while subtype C6 were identified to be associated with immune activity and MAPK pathway. Notably, miR-187 and miR-149 was identified to be targeted to the androgen-regulated gene in prostate cancer^35^,^36^. MiR-149 was also linked to the estrogen-receptor and progesterone-receptor signaling pathways in miRNA-seq subtype C3^37^. In miRNA subtype C6, lab results revealed that increased expression of miR-199 was associated with increased p38 MAPK activity and miR-143 and miR-145 could be blocked by p38 MAPK inhibitor^38^,^39^. These biological processes are also consistent with overlapped RNA-seq subtypes (Table 2).

For protein array and KEGG pathway, we found that two protein subtypes C1 ((P-value < 0.001), C2 (P-value < 0.01) and KEGG pathway subtype C4 (P-value < 0.001) significantly overlapped with RNA-seq subtype C9, which is associated with the activation of the MAPK signaling pathway. In addition, pathway cluster C1 (P-value < 0.01) and C2 (P-value < 0.05) overlapped with RNA-seq cluster C4, and pathway cluster C3 (P-value < 0.01) and C4 (P-value < 0.001) overlapped with RNA-seq cluster C5 (Fig. 3). We compared protein expression in their subtypes and KEGG pathway distribution with the RNA-seq subtypes (Fig. 2E,K and 3). We concluded that all of the subtypes associated with MAPK pathway activation have a relatively shorter survival duration. However, we found that there was one RNA-seq subtype that did not overlap any other omics subtypes, and this subtype was associated with immunoactivation. This result indicated that subtyping based on one omics dataset could not be fully replaced by other omics data.

## Discussion

The TCGA database provides an integrated perspective of ovarian cancer. We have described a comprehensive classification analysis of a large multicenter cohort of SOC that is correlated with the clinical outcome. Nine novel and robust SOC subtypes were identified using unsupervised clustering. We evaluated three unsupervised clustering algorithms: HC, PAM and NMF and found that, in most cases, the NMF model outperformed the other two models, indicating NMF is a more accurate and robust algorithm for cancer subtyping based on omics data.

Of greatest interest were the nine molecular groups that almost exclusively comprised the following four specific biological processes: immunoactivity, hormone metabolic, mesenchymal development and the MAPK signaling pathway. This result was consistent with previously published research in which the high-grade serous ovarian adenocarcinomas gene groups were differentiated, immunoreactive, mesenchymal and proliferative^8^. Richard W. Tothill *et al*. also reported that serous and endometrioid ovarian cancer are characterized by the stromal response, mesenchymal, immune signature, MAPK pathway and β-catenin/LEF/TCF complex^33^. C2, C4, and C6 were characterized by down regulation of hormone metabolic processes, the immune response and mesenchymal development, respectively. However, C7, C8, and C9 were characterized by over expression of mesenchymal development, immune response and the MAPK signaling pathway, respectively. C1 and C5 had dual characteristics. C1 was characterized by over expression of hormone metabolic and decreased expression of immune activity. However, C5 showed down regulation of mesenchymal development and the MAPK pathway. Interestingly, C3 was negative for all four. Meanwhile, we found that C8 had the best survival and C7 the worst survival, indicating that immune activity may have an additional benefit on survival, while mesenchymal development may be in contrast with the immune effect. Interestingly, we found most of the subtypes identified by RNA-seq were associated with the subtypes identified by other platforms, including promoter methylation, protein expression, miRNA expression, and signaling pathway analysis. We examined the association between the subtypes of different platforms in two different levels: patients and gene functions and we found the two levels were well consistent with each other.

Previously research has often provided a single platform. One example is the mutational spectrum. High-grade serous ovarian adenocarcinomas have prevalent TP53 mutations; mucinous ovarian cancer tumors have frequent KRAS mutations; and clear cell ovarian cancer and endometrioid ovarian cancer tumors have a lower rate of TP53 and frequent ARID1A and PIK3CA mutations^8^. Our research was has indicated that multi-omics can be used to describe the ovarian cancer profile. We also shed new light on some novel markers that mainly reflect the situation of gene expression. These gene markers have the potential to become novel hot spots of research and a new focus on pharmaceutical targets. Beyond acting as gene markers, the 16 survival-related proteins provide opportunities for further research. MiR-514 was reported in renal cell carcinoma to be related to recurrence and poor prognosis^32^, and it may be a potential specific marker for SOC.

We are dedicated to performing basic work to develop individualized treatment and precision medicine. One important further research topic is building prognostic models that incorporate clinical variables and multiple types of molecular data. In that regard, more effective treatment strategies should be developed for patients based on these models. Meanwhile, more specific drugs that target the novel genes and pathways should be developed. In addition, further efforts, particularly clinical trials and subsequent validations, are essential to evaluating our findings from the TCGA data.

## Methods

### Data source and variable selection

We downloaded clinical and molecular data (including DNA methylation, miRNA, RNA, and protein expression) from the TCGA Data Portal (https://tcga-data.nci.nih.gov/tcga/). There were 3 levels of molecular data. Level 1 included raw data, while levels 2 and 3 were processed data and normalized data, respectively. Specifically, these omics data from the following platforms were used and selected in our study (Table 1). For DNA methylation, level 3 was selected, which included whole genome methylation calling for each CpG site per sample. For miRNA, we chose both miRNA sequencing and array-based expression. For gene expression, we used RNA sequencing^8^,^9^. For protein expression, we downloaded the normalized protein expression for each gene in each sample. The Python programming language was used to extract data. Missing data (not available) and recurrent and normal solid tissue cohorts were deleted based on exclusion criteria. Only primary solid tumor sample was considered for analysis. Survival data from the patients are listed separately as a two-dimensional matrix. Each patient ID corresponded to the vital status (represented by 0 and 1 where 0 means survival and 1 means death) and survival days (if the patient was dead, the survival time was the number of days to survival, and if the patient survived, the survival time was the follow-up time, which was the correctly censored data)^40^. Moreover, a two-dimensional data matrix was built for all molecular and clinical data.

### Pathway activity calculation based on gene expression

We first converted the officinal gene symbol of all human genes to Entrez ID using the Gene ID Conversion Tool (DAVID bioinformatic resources 6.7). We then calculated the log-transformed fold change (logFC) of gene expression in each sample by dividing the expression level of each gene in this sample with the average expression level in all samples. We next used SPIA (Signaling Pathway Impact Analysis) in the R package, which implemented the SPIA algorithm to analyze KEGG signaling pathways^41^. The chosen pathways were dedicated to drawing tA values (the observed total accumulation value). The patient survival information binding with the tA values was assessed via cox regression analysis. We chose pathways with a statistically significant difference in survival. According to these selected pathways, patients were evaluated with unsupervised clustering and survival analysis.

### Gene functional enrichment analysis

We performed Gene Ontology^19^,^20^, KEGG pathway enrichment and Interpro (protein domain) analysis using the DAVID bioinformatic resources 6.7 toolkit (https://david.ncifcrf.gov/) for RNA-seq data and DNA methylation data. We performed miRNA target genes functional enrichment using the DIANA-mirPath v.3.0 web server with TarBase v.7.0 microRNA-target database^34^. Functional terms with *P*-value < 0.05 were treated as significantly enriched functions for each testing gene set.

### Implement of clustering algorithms

For DNA methylation, miRNA, gene and protein expression, we conducted a Cox regression analysis (the survival package in R) to identify molecular features that had a significant relationship with the patient survival. Using this method, we reduced the data dimensions from a variety of molecular data. DNA methylation and RNA were expressed by gene name and further analyzed by GO and KEGG. The miRNA and protein levels were analyzed by a literature review. For the selected dataset, unsupervised clustering was summarized using three methods, PAM, HC and NMF (NMF package R). We then conducted a survival analysis (survival package R) that combined a selected two-dimensional matrix with the patient survival. We tried to divide the data into 2 to 9 categories, which were used to compare the quality of clustering through the clustering score. The clustering score was defined as:


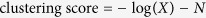


where X represents the P value of 2 to 9 clusters in each method and N corresponds to number of the cluster, from C2 to C9. The more categories in the penalty value, the greater its value. In the gene expression dataset, which was aimed at identifying the up-regulated and down-regulated genes in nine groups, we performed division operation to each set and the rest sets. We used the thresholds of 2-fold and 0.5-fold change to determine the up-regulated and down-regulated genes.

## Additional Information

**How to cite this article**: Zhang, Z. *et al*. Molecular Subtyping of Serous Ovarian Cancer Based on Multi-omics Data. *Sci. Rep.* **6**, 26001; doi: 10.1038/srep26001 (2016).

## Supplementary Material

Supplementary Table S1

Supplementary Table S2

Supplementary Table S11

## Figures and Tables

**Table 1:** Characterization platforms used and data generated.

Data type	Platforms	Cases	Level
DNA methylation	Illumina Infinium Human DNA Methylation 27	583	Level 3
Protein	MD Anderson reverse phase protein array	412	Level 3
MiRNA	Agilent Human miRNA Microarray Rel12.0	570	Level 3
	Illumina Genome Analyzer miRNA Sequencing	475	Level 3
RNA	Illumina HiSeq 2000 RNA Sequencing	422	Level 3

**Table 2:** Nine SOC subtypes across the four biological processes and MUC gene family.

Subtypes	Immuno-activity	Hormone metabolic	Mesenchymal development	MAPK pathway	MUC family
C1	Down	Up	−	−	−
C2	−	Down	−	−	−
C3	−	−	−	Down	−
C4	Down	−	−	−	−
C5	−	−	Down	Down	−
C6	−	−	Down	−	Up
C7	−	−	Up	−	−
C8	Up	−	−	−	−
C9	−	−	−	Up	−

**Table 3:** Analysis of clinical variables related to gene expression.

Clinical variables	F value/X-squared	P-value
Intermediate dimension	0.659†	0.728
Longest dimension	0.756†	0.641
Shortest dimension	0.771†	0.629
New tumor event diagnosis days	1.852†	0.072
Ecog score	1.253†	0.279
Karnofsky score	0.474†	0.867
Tumor grade	1.486†	0.169
Clinical stage	4.786^†^	1.22 × 10^−5^***
New tumor event diagnosis evidence	35.141‡	0.322
New tumor event radiation treatment	4.876‡	0.771
New neoplasm event type	35.491‡	0.307
Pharmaceutical treatment adjuvant	7.03‡	0.533
Treatment outcome first course	38.671‡	0.03*
Tumor status	17.894‡	0.022*
Residual disease largest nodule	40.183‡	0.02*
Vascular invasion indicator	7.569‡	0.477
Lymphovascular invasion indicator	7.082‡	0.528
Anatomic neoplasm subdivision	17.604‡	0.348

^†^These are quantitative variables evaluated with ANOVA for statistical analysis. The test statistic is the F value.

^‡^These are qualitative variables evaluated with the chi-square test. The test statistic is X-squared.

^*^P < 0.05.

^**^P < 0.01.

^***^P < 0.001.
